# Reduced Cerebral Glucose Uptake in an Alzheimer’s Rat Model With Glucose-Weighted Chemical Exchange Saturation Transfer Imaging

**DOI:** 10.3389/fnagi.2021.618690

**Published:** 2021-03-17

**Authors:** Peidong Chen, Zhiwei Shen, Qianqian Wang, Bingna Zhang, Zerui Zhuang, Jiefen Lin, Yuanyu Shen, Yanzhi Chen, Zhuozhi Dai, Renhua Wu

**Affiliations:** ^1^Department of Medical Imaging, The Second Affiliated Hospital, Shantou University Medical College, Shantou, China; ^2^Philips Healthcare, Beijing, China; ^3^Department of Postgraduate, Shantou University Medical College, Shantou, China; ^4^Center for Translational Medicine, The Second Affiliated Hospital, Shantou University Medical College, Shantou, China

**Keywords:** Alzheimer’s disease, glucoCEST, glucose metabolism, D-glucose, magnetic resonance imaging

## Abstract

A correlation between the abnormal cerebral glucose metabolism and the progression of Alzheimer’s disease (AD) has been found in previous studies, suggesting that glucose alterations may be used to predict the histopathological diagnosis in AD. In this study, we investigated the dynamic changes of cerebral glucose uptake *in vivo* using MR glucose chemical exchange saturation transfer (glucoCEST) imaging in a rat model of AD with an intracerebroventricular (i.c.v) injection of amyloid Aβ-protein (25–35), confirmed by Morris water maze and Nissl staining. In total, 6 rats in the AD group and 6 rats in the control group that were given an injection of sterile normal saline were included. At 28 days after injection, all rats performed a 7.0 T MR exanimation, including glucoCEST, diffusion tensor imaging (DTI) and hippocampus magnetic resonance spectra (MRS), to detect the possible metabolic and structural changes in the rat brain. A significantly elevated brain glucoCEST signal in the brain of AD rats was observed, and a decreased brain glucose uptake was also explored during the progression of glucose infusion compared with those in rats of the control group. In addition, there is a significant positive correlation between glucoCEST enhancement (GCE) and myo-Inosito (Ins) in the AD group and the control group (*P* < 0.05). A significantly reduced number of neurons in the cortex and hippocampus in AD rats combined with the significantly longer escape and a decreased number of crossings were verified at 28 days after Aβ25–35 injection by Nissl staining and Morris water maze, respectively. Our results indicated that an abnormal brain glucose mechanism in AD rats could be detected by glucoCEST imaging, suggesting a new method to explore the occurrence and progress of diabetes-related AD or dementia.

## Introduction

Alzheimer’s disease (AD) is a chronic progressive neurodegenerative disease characterized by the accumulation of amyloid-β protein and a Tau-meditated neuronal injury (de Paula et al., 2009; de-Paula et al., 2012). AD affects around 50 million persons worldwide, and the number will likely increase with longer life expectancy (Abbott, 2011). Clinically, patients with AD are presented with a combination of features, such as progressive cognitive decline, dementia, and personality changes (Jack et al., 2013). The deposition of amyloid-β peptides has been observed in AD animal models as well as post-mortem AD patients, suggesting amyloid-β as having a key role in AD pathogenesis (Seynnaeve et al., 2018). Nevertheless, accumulated evidence shows that the pathogenesis of AD is also contributed to by multiple key players (de-Paula et al., 2012).

Glucose is a primary source of energy for almost all living organisms. Impaired glucose metabolism is associated with a wide range of pathological conditions (Mergenthaler et al., 2013) and is considered an important risk factor for AD (Duran-Aniotz and Hetz, 2016). The reduction of glucose uptake in posterior cingulate and temporal-parietal regions is the most commonly described diagnostic criterion for AD (Wang et al., 2016) with 18F-fluorodeoxyglucose (FDG) positron emission tomography (PET), and it was considered to be associated with the cognitive decline in healthy control (Gardener et al., 2016). Moreover, type 2 diabetes was shown with an increased risk of cognitive decline and developing AD (Mergenthaler et al., 2013). Therefore, the interplay between glucose metabolism and AD should be better understood, especially during the early stages of AD.

Over the last three decades, 18-FDG PET has provided important information for the diagnosis and differential diagnosis of AD in animal models and patients. Le Douce et al. (2020) found that there was a decreased glucose uptake in the amygdala, entorhinal cortex and hippocampus in 3xTg-AD transgenic mice in the early phase with 18F-FDG PET. In patients with AD, reduced glucose metabolism was also observed by 18F-FDG-PET in the temporal and superior temporal/posterior temporal regions, posterior cingulate cortex and the anterior wedge (Herholz, 2010; Kantarci et al., 2010). However, contradictory results were also found in APP/PS1 transgenic mice, indicating an increased glucose utilization in multiple brain regions at 2 and 3.5 months of age (Li et al., 2016).

Chemical exchange saturation transfer (CEST) imaging is a relatively new MR contrast technique and has been used to map exchanged protons or molecules in glucose, amide proton, glutamate or creatine *in vivo*. Based on a frequency-specific saturation pulse, the magnetization transfer effect occurred among bulk water and labile solute protons in the above metabolites and further lead to a decreased signal intensity of free water. By detecting the changes of the free water signal, the effect of glucose chemical exchange saturation transfer (glucoCEST) can be observed, which is related to the concentration of glucose and exchange environments, such as pH and temperature. GlucoCEST is considered a non-invasive alternative for the *in vivo* imaging of glucose uptake following an external administration of glucose (McMahon, 2017). Moreover, glucoCEST enhancement (GCE) imaging was achieved by subtracting the difference glucoCEST images before and after glucose administration, which is similar to dynamic contrast-enhanced (DCE) MR perfusion by the injection of gadolinium-based contrast agent. Using glucoCEST, glucose uptake in healthy mice brains (Nasrallah et al., 2013), colorectal tumor models (Walker-Samuel et al., 2013) and healthy mice livers (Miller et al., 2015) was successfully detected. In this study, we aim to explore the dynamic changes of cerebral glucose uptake in AD model rats using both glucoCEST and GCE. The possible pathological and behavioral changes in the rat brain of the AD group were detected by Nissl staining and a morris water maze to confirm the AD model. Furthermore, the correlation between glucose metabolism, diffusion characteristics and several hippocampus metabolites was investigated for the interpretation of abnormal glucose metabolism.

## Materials and Methods

### Animals

A total of 12 male Sprague-Dawley (SD) rats (between 16 and 18 weeks of age and weighing 250–320 g) were purchased from the Animal Center Laboratory of our Medical College. The rats were randomly divided into the control group (*n* = 6) and the AD group (*n* = 6). All rats were housed in plastic cages at 12 h light/dark cycle with free access to food and water. All experiments were approved by our ethics committee of Animal Care and Welfare.

### AD Rat Modeling

Amyloid β-Protein Fragment 25–35 (Aβ25–35; Sigma-Aldrich) was dissolved in sterile saline at a concentration of 1 mg/ml and stored at −20°C till to be used. Before injection, Aβ25–35 was aggregated in an electronic thermostat at 37°C for 4 days (D’Agostino et al., 2012; Kim et al., 2015).

With reference to the method described by previous studies (Ohta et al., 2012; Kim et al., 2015), the AD rat model was established. Briefly, rats were anesthetized with an intraperitoneal injection of pentobarbital sodium (40 mg/kg) and placed in a stereotaxic device. Following midline incision, a dental drill was used to perforate skull over the right lateral ventricle at the coordinates (0.8 mm posterior to the bregma and 1.5 mm lateral to the midline). Rats in the AD group were transfused with 9 μl of aggregated Aβ 25–35 into the right lateral ventricle using a microsyringe at a rate of 1 μl/min, and the needle was then left in place for an additional 5 min to allow adequate diffusion. Meanwhile, the rats in the control group were injected with sterile normal saline.

### Estimation of Blood Glucose Concentration

On the 14th day after the intracerebroventricular injection of Aβ25–35, three rats in the AD and control groups were randomly selected to acquire the time-blood glucose concentration curves by continuous injection of D-glucose infusion according to the method described previously (Chan et al., 2012). The rats were anesthetized by 1.5% isoflurane in oxygen gas during the whole experimental procedure. D-glucose (1.5 mM) was dissolved in normal saline and was continuously injected using a microsyringe pump (JMS, Japan, model SP-500) at a rate of 2.0 ml/h. The blood glucose concentration was examined in a sample obtained from the tail vein at the following time points: before injection, 10, 20, 30, 40, and 50 minters after injection and 10 and 20 minters after the finish of glucose infusion.

### Optimization Scan Parameters of GlucoCEST Imaging in Phantom

Firstly, seven centrifugal tubes containing the following different metabolites were scanned to acquire an optimized saturation power referenced with the method detailed previously (Haris et al., 2013). The metabolites in each tube included creatine (Cr, 10 mM), glutamate (glu, 10 mM), gamma-aminobutyric acid (GABA, 10 mM), choline (cho, 10 mM), high D-glucose concentration (200 mM), low D-glucose concentration (10 mM) as well as D-glucose and Cr mixture (5 mM each), respectively. Next, another six tubes containing D-glucose with different concentrations (6.25, 12.5, 25, 50, 75, and 100 mM) in pH of 7.4 were scanned to observe the CEST exchange signal affected by the concentration (Nasrallah et al., 2013). Finally, three tubes containing D-glucose (25 mM) with different pH (6.4, 7.4, and 8.4) were examined to observe the impact of pH on the glucose CEST exchange. All phantoms (Haris et al., 2012) were prepared in phosphate buffer solution (PBS) supplemented with 1% agarose solution.

Several important scan paraments of glucoCEST imaging, such as saturation power, saturation during and repetition time (TR), were optimized in phantom model with varying values: saturation power (1.5, 3.0, and 6.0 μT) in the tubes with different metabolites; saturation power (1.5, 2.0, 2.5, 3.0, 3.5, and 4.0 μT) in the phantoms with different concentration D-glucose; saturation during (4, 5, and 6 s) and TR (5, 6, 7, and 8 s), respectively (Nasrallah et al., 2013; Dai et al., 2014). In addition, the phantom model with different pH D-glucose concentrations was scanned with B1 = 1.5 μT, saturation time 5 s.

### MRI Acquisition in Phantom and Animal Examination

In the phantom test, an echo-planar imaging (EPI) sequence with a continuous wave saturation pulse was used to perform glucoCEST imaging to explorer the optimized scan parameters. The acquisition parameters were set as the following: TR 5.04 s, slice thickness 2 mm, acquisition matrix 64 × 64, field of view of 40 mm × 40 mm, averages 1, pre-saturation during 5 s, the saturation offset range from –1,500 to +1,500 Hz, the step 30 Hz and reference image without the saturation offset of 10,000 Hz. A B0 and B1 map were collected to correct the possible asymmetry and variation of magnetic field.

All rats were performed MR imaging at 28 days after the intracerebroventricular injection with Aβ25–35/sterile normal saline in the AD group/control group. Before MR examination, all rats were allowed to fast for 24 h. The rats were firstly anesthetized in an induction box with 4% isoflurane in oxygen and continued to inhale the gas mixture of 1.5%/98.5% isoflurane/oxygen via a nose tube during the MRI scanning (Tang et al., 2017). The respiration rate was monitored and maintained by animal physiological monitoring and gating systems (model 1030, SAII, United States). Then, rats were fixed on an MRI matching sampler with a bite and two ear bars to prevent the head motion.

MR imaging in phantom and animal were performed on a 7.0 T animal MR scanner (7T/160/AS Agilent Technologies, United States) with a 9,563 volume transmit/receive coil. The protocol included Axial and sagittal T2-weighted, DTI, and bilateral hippocampal MR spectroscopy and glucoCEST imaging. Axial and sagittal T2W imaging was acquired to demonstrate the morphology of rats’ whole brain, and the parameters were set up as follows: TR 2,000 ms, acquisition matrix 256 × 128, field of view 40 × 40 mm, slice thickness 2 mm, slice 6, slice spacing 0.2 mm and average 2. With the reference to T2WI, the scan layer including the middle hippocampus was selected as the area of interest. Axial Diffusion Tensor Imaging (DTI) of the rat brain was obtained with the following parameters: the scanning layer is consistent with glucoCEST imaging, TR 2,000 ms, TE 39.68 ms, FOV 40 mm × 40 mm, slice thickness 4 mm, matrix 128 × 128, b 1031.0 s/mm^2^, and the average 8. A total of seven diffusion-weighted images with S0 and six directions were obtained (b = 1031.0 s/mm^2^) in a total scan time of 10 min and 41 s. Then, 1-H Magnetic Resonance Spectroscopy (MRS) was performed in the bilateral hippocampus before D-glucose infusion using a Point RESolved Spectroscopy (PRESS) pulse sequence with the following parameters: TR 4,000 ms, TE 13 ms, averages 192, and a spectral width of 4,006 KHz.

The axial glucoCEST images of the rat brain were acquired before the intraperitoneal injection of D-glucose (1.5 mM) and 10, 20, 30, and 40 min during infusion and then 10 and 20 min following the infusion. The scan parameters were: TR 6 s, slice thickness 4 mm, acquisition matrix 64 × 64, field of view of 35 × 35 mm^2^, average 1, saturation during of 5 s, saturation offset range from –900 to +900 Hz at intervals of 30 Hz and magnetization references at 10,000 Hz. The saturation power of 1.5 μT was used with the minimize influence of other metabolites on glucoCEST and direct water saturation effect.

### Morris Water Maze

After MR examination, a Morris water maze (MWM) was used to evaluate learning and memory dysfunction. MWM experiments were carried out on the 14th and 28th day following Aβ25–35 administration as described previously (D’Agostino et al., 2012; Ohta et al., 2012). Rats were trained for 4 days (4 trails/day), and the swimming pattern was captured with a camera and a video track software (Noldus, Co., Ltd. Holland) for analysis. The latency to reach the platform was acquired using a computer-controlled tracking system. Rats were allowed 120 s to reach the platform then they were guided to the platform manually and left there for 5 s. On the fifth day, the platform was removed and a 120 s-spatial probe-trial was performed. The number crossing the platform position, the time of crossing from the target quadrant and the swimming speed of each rat were recorded.

### Nissl Staining

Finally, rats were sacrificed and underwent myocardial perfusion with saline followed by 4% paraformaldehyde. The brains were removed and fixed in 4% paraformaldehyde overnight. Next, the brain tissues were dehydrated in ethanol, embedded in paraffin, subjected to coronal sectioning into 5 μm thickness and required to undergo Nissl staining (Beyotime Co., Ltd., Shanghai, China) according to the standard protocols. The Nissl staining results were observed by an uninformed examiner. Three consecutive but non-overlapping fields of vision (magnification of 40×) in the hippocampal CA1 region and cerebral parietal cortex were randomly selected from each rat brain tissue, and the neurons were counted with Image J software and showed as mean values per high-power field (HPF).

### Data Analysis

All glucoCEST images were processed using a custom MATLAB (The Mathworks, Inc., Natick, MA, United States) routine. Regions of interest (ROIs) were drawn manually based on the T2-weighted images. Total brain, bilateral hippocampal and cortex were drawn as ROI to observe the abnormal GCE in AD rats. After B0 correction, Z-spectra were obtained by the ratio of the signals from different irradiation frequency offsets and the signal of water protons without saturation (S0). The magnetization transfer ratio asymmetry (MTRasym) was calculated as MTRasym (0.9 ppm) = [Ssat (−0.9 ppm) −Ssat (+0.9 ppm)]/S0. Meanwhile, the GlucoCEST enhanced (GCE) images were calculated as the subtraction of the glucoCEST image before D-glucose injection and the mean glucoCEST images of 40 min after injection and 10 min after the finishing D-glucose injection.

1-H MRS data were analyzed with LCModel software (version 6.3, LCModel company, Canada) to acquire the concentrations of metabolites, such as N-acetyl aspartate (NAA), glutamate (Glu), glutamine (Gln), creatine (Cr), taurine (Tau), myo-Inosito (mI), glutathione (GSH), NAA + N-acetylaspartylglutamate (NAAG), glycerophosphocholine + phosphocholine (GPC + PCh), Glu + Gln, and Cr + phosphocreatine (PCr). A standard deviation (SD) value of less than 20 was accepted as being indicative of more reliable data.

In diffusion imaging, a brain apparent diffusion coefficient (ADC) map and fractional anisotropy (FA) map was acquired by VnmrJ (version 4.0, Agilent Technologies, United States) and subsequently analyzed by MATLAB software to calculate the ADC and FA in the total brain.

### Statistical Analysis

All data were analyzed statistically using SPSS 20.0 software (IBM, United States). Taking the small sample size in this study into consideration, outlier detection was firstly performed with a stem-and-leaf plot. Then normal distribution and homogeneity of variance were verified by a Shapiro-Wilk test and Levene’s test, respectively. If a normal distribution and variance homogeneity tests were satisfied simultaneously, the group differences were analyzed with an independent sample *t*-test. Otherwise, a non-parametric test was used. In MWM data, square-root transformation was used to fit normal distribution and homogeneity of variance. One-way ANOVA repeated measures were performed to detect the group differences of escape latency over 4 days. The correlation among glucose metabolism and other metabolites in bilateral hippocampal were assessed using linear regression analysis. A *p* < 0.05 was considered to be a statistically significant difference.

## Results

### Optimization of Scan Parameters in Phantom

**FIGURE 1:**
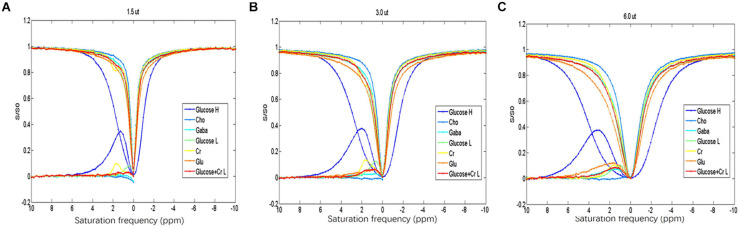
**(A–C)** Z-spectrum and MTR of the different metabolites indicating that D-glucose can be distinguished from the other metabolites at saturation energy B1 of 1.5 μT.

Among the different metabolites, the CEST effect of D-glucose could be better distinguished at a saturation power of 1.5 μT (Figure 1A). The overlap of CEST effects of different metabolites is more serious with the increased saturation power from 1.5 to 6 μT (Figures 1B,C).

**FIGURE 2:**
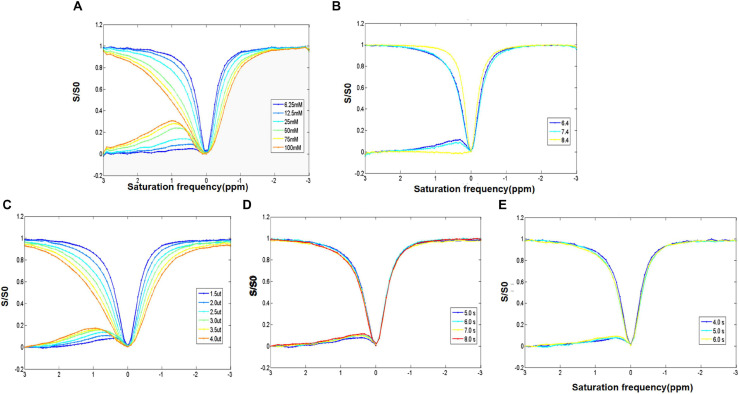
**(A)** Z-spectrum and MTR of different concentrations of D-glucose showing the direct saturation exchange rate of D-glucose and that water protons increase with the increase of D-glucose concentration. **(B)** Z-spectrum and MTR of D-glucose with different PH values examining the impact of pH. It indicates that the D-glucose effect is negatively correlated with pH. **(C–E)** Z-spectrum and MTR investigating the optimization of the saturation energy and echo and saturation time parameters. The D-glucose CEST effect increases with the increase in saturation energy and saturation and echo times.

The increase of D-glucose concentration resulted in the broadening of the glucoCEST signal in the asymmetric spectrum curve in the range of 0.5–1.0 ppm (Figure 2A). Moreover, a shift of the peak of D-glucose asymmetric spectrum curve (from 0.5 to 1.3 ppm) was found, and the D-glucose concentration (25–100 mM) was positively associated with the glucoCEST effect (10–25%) (Figure 2A).

A negative correlation was observed between the pH and the glucoCEST exchange rate. A decreased glucoCEST asymmetry signal was observed with the increase of pH value (Figure 2B). At a pH of 7.4, the glucoCEST exchange rate was about 10%. With the increase of saturation power from 1.5 to 4.0 μT, there was a gradual rise in MTRasym (0.9 ppm), and the peak of the asymmetry curve shifted to a higher frequency (Figure 2C). Elevated MTRasym (0.9 ppm) (7.5, 9, 9.5, and 11.5%, respectively), was found with increasing repetition times (5, 6, 7, and 8 s); however, the scan time was also significantly increased (Figure 2D). Meanwhile, with an increase of the saturation duration from 4 to 6 s, MTRasym (0.9 ppm) increased from 7 to 11% (Figure 2E) with a prolonged scan time. Taken the scan time and MTRasym (0.9 ppm) into consideration, the optimal scan parameters of the glucoCEST were as follows: pH of 7.4, TR of 5.04 s, saturation power of 1.5 μT and saturation during of 5 s.

### Decreased Brain GCE Signal in AD Rats

**FIGURE 3:**
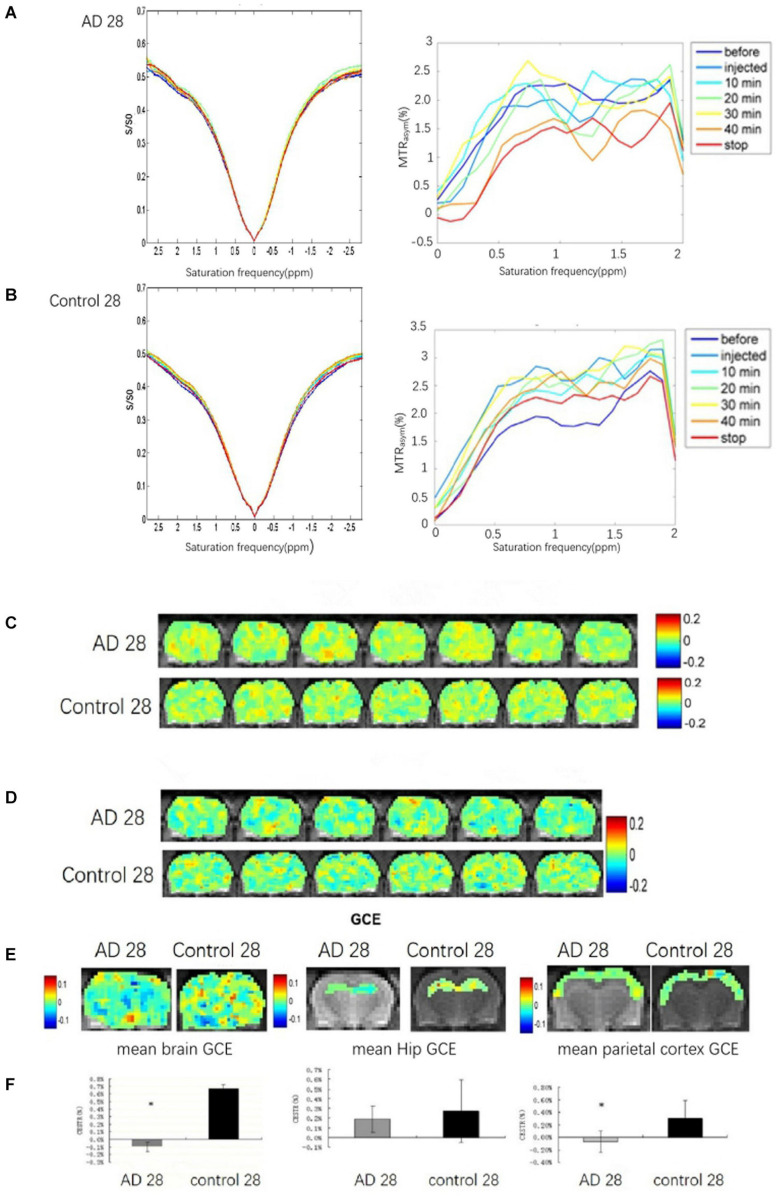
The brain GCE signal of a rat in AD and control groups at 28 days after Aβ25–35 administration, respectively. **(A,B)** Showing Z-spectra and MTR_asym_ of AD and control rat brains. **(C,D)** Showing glucoCEST and GCE images of AD and control rat brain at 28 days after Aβ25–35 administration in specific time point, respectively. **(E)** The mean GCE signal of the total brain, hippocampus, and parietal cortex in an AD rat and a healthy rat. **(F)** The corresponding bar chart to the mean GCE signal of the total brain, hippocampus and parietal cortex in AD and control rats. It indicates that the glucose uptake of the whole brain in AD rats was significantly decreased, *P* < 0.05; the glucose uptake of the AD rats hippocampi is reduced, but *p* > 0.05; the glucose uptake of the parietal cortex in AD is decreased, *p* < 0.05 (*p < 0.05, error bar was standard error).

The brains glucoCEST asymmetric spectrum and Z-spectrum of all rats in the AD group and the control group were detected at 28 days after Aβ25–35 administration (Figures 3A,B). The lowest brain GCE in AD rats was observed at 40 min and after stopping the infusion particularly in the thalamic and the hippocampal regions (Figures 3C,D). Additionally, in control rat brains, the change of GCE signal showed homeostasis without a downward trend.

Because of abnormally elevated GCE signal, one case in the AD group was excluded. Compared to the control rats (*n* = 6), significant decreases in the GCE signal of total brain and AD rats (*n* = 5) were observed at continuous injection for 40 min and at 10 min after the stop of the injection (*P* < 0.05; Figures 3E,F). The decreased GCE signal was also found in the bilateral parietal cortex of AD rats with significant statistical differences (*P* < 0.05; Figures 3E,F), but there were no significant statistical differences in the bilateral hippocampus (*P* > 0.05; Figures 3E,F).

### Metabolite Concentrations in the Bilateral Hippocampal

**FIGURE 4:**
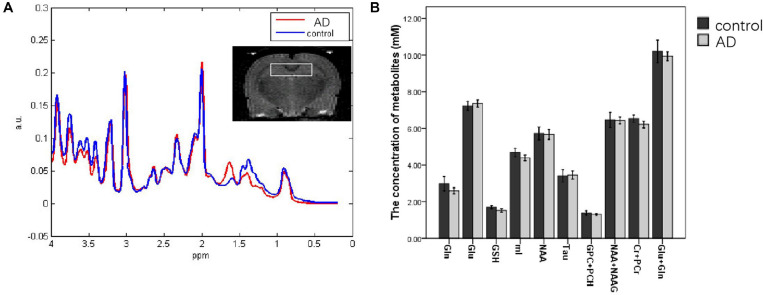
MRS images analyzing the metabolite content in the hippocampi of control and AD rats at 28 days after Aβ25–35 administration **(A)**; the white box in the rat brain is the volume of interest of MRS scanning. **(B)** Metabolite concentration in the hippocampus at 28 days after Aβ25–35 administration; it showed no difference was found between the two groups (all *P* > 0.05, error bar was standard error).

One case in the control group was cut off because of the more than 20 metabolites in the SDs. Compared to the control group (*n* = 5), a descending trend of the concentrations from bilateral hippocampal in AD rats (*n* = 5) was observed in the Gln, mI, Cr + PCr, and Glu + Gln at 28 days after Aβ25–35 administration. However, there was no significant difference in the concentrations of all metabolites between the AD group and the control group (*P* > 0.05; Figure 4).

### The Correlation Between mI Concentration and GCE Signal

**FIGURE 5:**
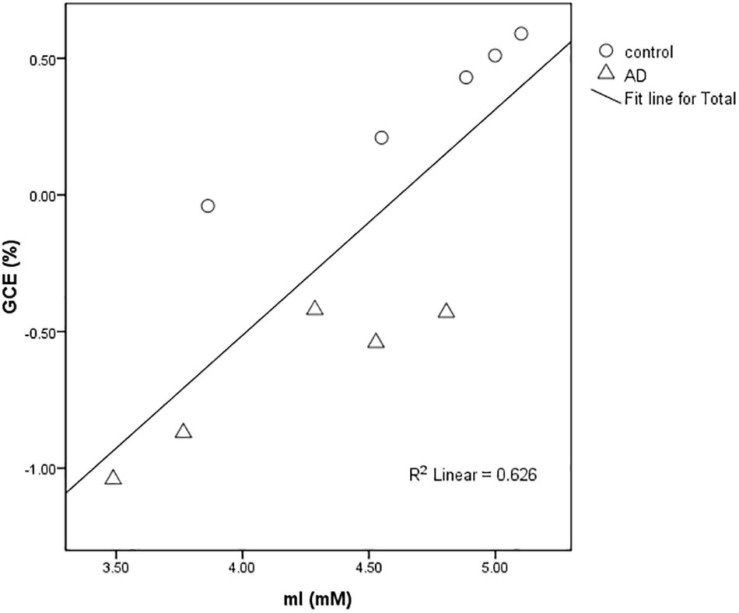
Scatter plot of the relationship between the mean GCE signal of rat total brain and hippocampal MI concentration detected by MRS in the AD group and control group at 28 days after Aβ25–35 administration. The graph showed a positive correlation between MI concentration and GCE signal (*R*^2^ = 0.626).

In rats of the control group (*n* = 5) and the AD group (*n* = 5), a strong positive correlation between GCE and mI (*R*^2^ = 0.626) was found with significant statistical differences (*P* < 0.01) at 28 days after Aβ25–35 administration (Figure 5).

### Comparison of ADC and FA of Total Brain

**FIGURE 6:**
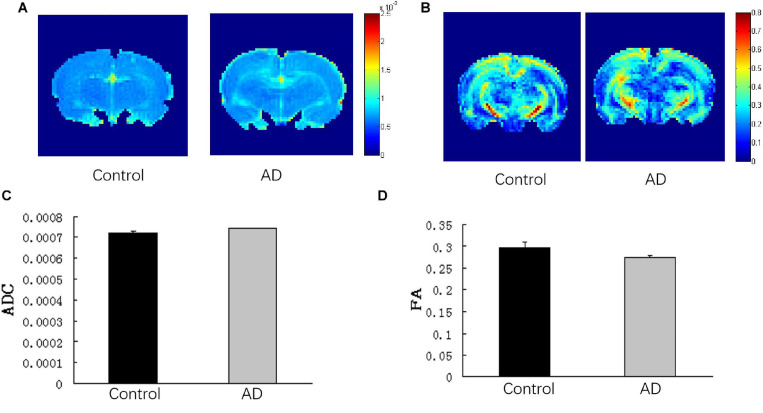
Analysis of DTI imaging of rat brains in the AD group and control group at 28 days after Aβ25–35 administration. **(A)** ADC images and **(B)** FA images of the control and AD rat brains. **(C)** Bar chart demonstrating the corresponding ADC value of the control and AD rats. *P* > 0.05. **(D)** Bar chart representing the corresponding FA value of the control and AD rats. *P* > 0.05, error bar was the standard error (*n* = 6 per group).

There was no significant difference in the ADC values and FA values of the total brain between the AD and control groups (*P* > 0.05; Figure 6).

### Impaired Spatial Learning and Memory in AD Rats

**FIGURE 7:**
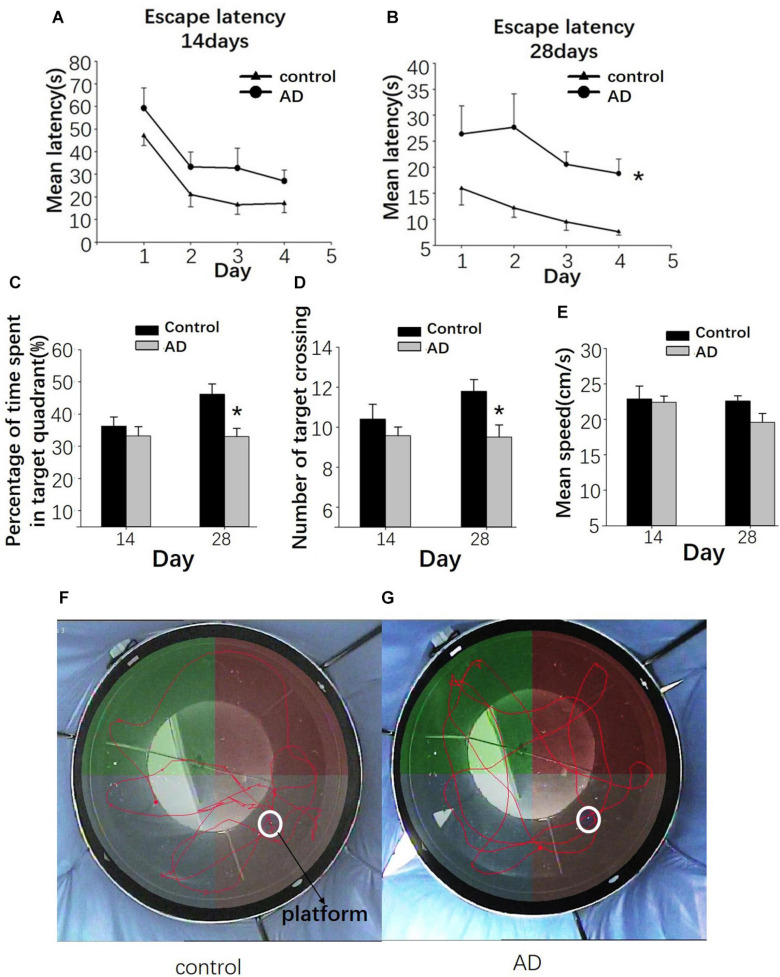
Evaluation of the learning and memory dysfunction in rats of the control and AD groups at 14 and 28 days after Aβ25–35 administration by Morris water maze (MWM). The escape latency of control and AD rats **(A,B)**. **(C)** Bar charts presenting the percentage of target quadrant crossings. **(D)** Bar charts presenting the number of target crossings. **(E)** Bar chart presenting the analysis of swimming speed among rats of the control and AD groups. **(F)** Tracing of the swimming pattern in the control rats. **(G)** Tracing of the swimming pattern of the AD rats. *n* = 6 per group, *P < 0.05, error bar was the standard error.

At 14 days after Aβ25–35 administration, the escape latency was relatively longer in the AD rats than in the control group; however, the difference was not statistically significant (*P* > 0.05; Figure 7A). At 28 days after Aβ25–35 injection, a significantly longer latency to find a platform in the AD rats was found compared to those in the control rats (*P* < 0.05; Figure 7B), indicating a slower learning pattern in the AD rats. Furthermore, fewer crossing times to the platform and the time to cross the target quadrant in AD rats were observed at the 28 days following Aβ25–35 administration, and they were statistically significant (*P* < 0.05; Figures 7C,D,F,G), though no significant difference in the average swimming speed was found between two groups (*P* > 0.05; Figure 7E).

### Decreased Number of Hippocampal CA1 and Parietal Nissl Body in AD Rats

**FIGURE 8:**
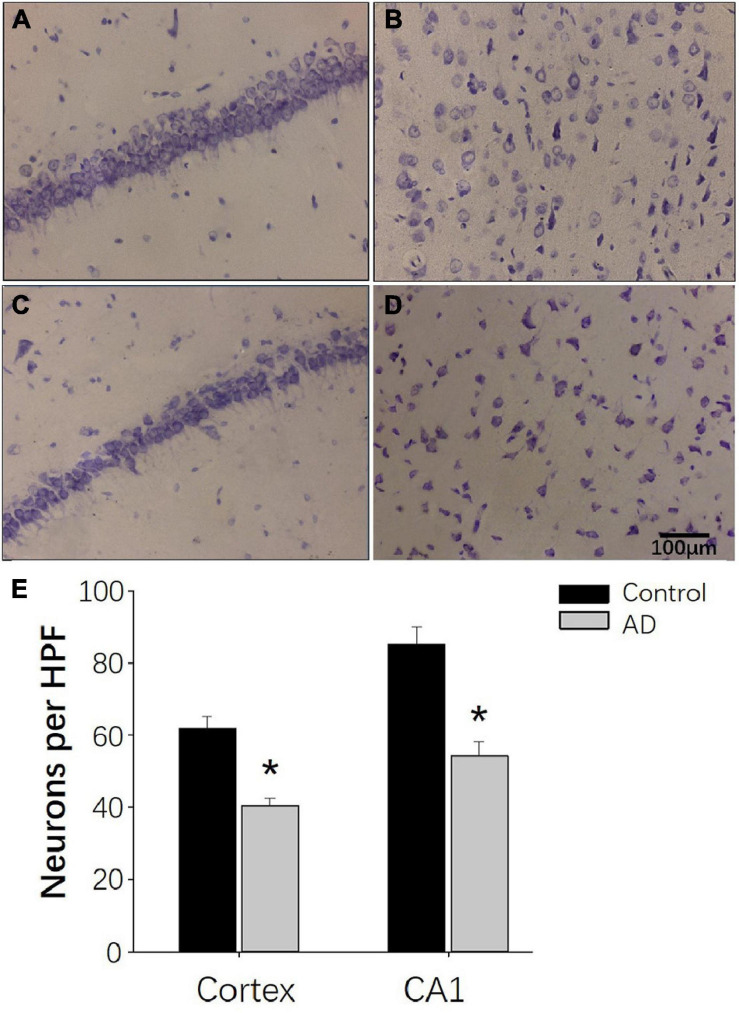
Nissl staining demonstrating the decreased neurogenesis in AD rats at 28 days after Aβ25–35 administration. **(A,B)** Representative photomicrographs of the hippocampal CA1 region **(A)** and the internal pyramidal layer of parietal cortex **(B)** in control rats demonstrating normal neural cytoarchitecture. **(C,D)** Representative photomicrographs of the hippocampus CA1 region **(C)** and the internal pyramidal layer of the parietal cortex **(D)** in the AD group, demonstrating reduced numbers of neurons and neural damage. **(E)** Bar chart presenting the number of neurons per HPF in the internal pyramidal layer of parietal cortex and hippocampal CA1 area of AD and control group. *n* = 6 per group, *p < 0.05, scale bar = 100 μm.

Nissl staining was used to observe the neural loss and damage in the AD rat brains after 28 days of Aβ25–35 administration. Rats in the control groups showed typical Nissl staining in the hippocampus with deeper staining, regular and tighter neural arrangement and normal cytoarchitecture (Figure 8A). In the AD group, the hippocampal neurons had reduced numbers of Nissl bodies, lighter staining, irregular cell arrangement in the CA1 region with intact cell structure and blurred cell nuclei (Figure 8C). Similar observations were found in the cerebral cortex (Figures 8B,D). In the control group, Nissl bodies were observed in the cortical nerve cells with typical deep staining and normal cytoarchitecture (Figure 8B). In the AD group, the number of Nissl bodies in the cortical neurons was decreased with a shallow staining and abnormal cytoarchitecture (Figure 8D). Otherwise, compared to the control group, the numbers of neuronal cells per HPF of hippocampal CA1 and parietal cortex neurons in the AD group were decreased (*P* < 0.05; Figure 8E).

## Discussion

In this study, we investigated the changes in the cerebral glucose metabolism with GlucoCEST in a rat model of AD. A significantly decreased mean GCE signal of total brain and bilateral parietal cortex of AD rats was observed after 40 min of continuous injection of D-glucose and 10 min after the stop of injection. Moreover, there is a strong positive correlation between GCE and mI in the rats of the control group (*n* = 5) and the AD group (*n* = 5), suggesting the presence of abnormal glucose metabolism in the brain of AD rats.

Different from previous animal AD studies with transgenic mice models, we chose the AD rat models for acquiring the brain glucoCEST images with high SNR. The method of AD rat modeling is based on a previous study (Lu et al., 2009), and the water maze test and pathological examination were performed to verify the abnormal behavioral and pathological changes in rats with the intracerebroventricular injection of aggregated Aβ25–35 (9 μl). The injection of 9 μl Aβ25–35 was demonstrated to cause severe neurotoxicity leading to impaired learning and memory function, the release of inflammatory mediators and impairment of neuronal cells (Lu et al., 2009). Indeed, in our study, rats in the AD group had a reduced number of neurons in the cortex and the hippocampus with altered neural architecture after Aβ25–35 injection. These results are consistent with previous studies (Maurice et al., 1996). Moreover, AD rats had significantly longer escape latency and a decreased number of crossing times compared to rats in the control group at 28 days after the injection of Aβ25–35. These results indicate that the capacity for space exploration was impaired in AD rats, and the same was true for their learning and memory abilities. Taken together, our results confirm the feasibility of single Aβ25–35 administration in inducing AD symptoms in rats (Kim et al., 2015).

D-glucose is a glucose metabolite produced by the body’s metabolism that can pass through the blood–brain barrier (Nasrallah et al., 2013). GlucoCEST is based upon the fact that each glucose molecule contains five hydroxyl groups that can be chemically exchanged with hydrogen protons in the free-water molecule (Walker-Samuel et al., 2013). Consequently, this reduces the strength of the MRI free-water signal that can reflect the content of D-glucose. Therefore, D-glucose can be used as an MRI contrast agent to increase the imaging sensitivity. Walker-Samuel et al. (2013) indicated the presence of an intracellular origin of glucoCEST using D-glucose, and Nasrallah et al. (2013) reported that most of the glucoCEST signals seen in the brain after injecting 2DG are of an intracellular origin. In this study, the GCE technique was used to detect the uptake of exogenously administered D-glucose in the brain.

Previous studies used glucoCEST to examine the glucose metabolism in tumors, and the brain sugar content was mainly examined with ^18^FDG–PET (Coleman et al., 2017). In the few AD relative studies using glucoCEST imaging, Wells et al. (2015) explored the glucose metabolism in AD and found that elevated CEST signal in the cortex of AD mouse, which is consistent with our results. But the quality of glucoCEST images in Wells’ study needs to be improved. In this study, an EPI sequence with a continuous-wave saturation pulse was used, and it had been proven to be able to improve the CEST contrast ratios and the quality of glucoCEST images (Sun et al., 2008). Furthermore, the important scan parameters of glucoCEST imaging, such as saturation power, saturation duration and TR were optimized in phantom to improve the quality of brain glucoCEST images.

The glucose tolerance test was performed in fasted rats in this study using intraperitoneal glucose injections. To prolong the time window of blood glucose peaks and maintain a higher concentration of glucose in the brain, we used a syringe pump to continue the small dose supplementation. In the control group, the brain GCE signal continued to rise and was consistent with the trend of blood glucose monitoring, confirming the reliability of our method. The GCE signals, however, were significantly reduced in the AD group, and these results were in accordance with the previously reported ^18^FDG–PET studies (Waldron et al., 2015; Coleman et al., 2017).

In glucoCEST images before D-glucose injection, we found more elevated MTRasym (0.9 ppm) signals in the total brain of AD rats than in the control group. Except for technical issues such as B0/B1, direct water saturation and MT, the CEST signal is considered related to the concentration of exchanged protons or molecules, T1 relaxation time of tissues and the exchanged environment, including pH and temperature. Although elevated glucose/Cr in the AD brain was reported (Mullins et al., 2018), the effect of other issues was not excluded. Further experiments are thus needed to confirm the elevated MTRasym (0.9 ppm) signal mainly comes from elevated glucose concentration. As for the molecular mechanism of abnormal glucose metabolism and AD, the insulin receptor substrate 2 (IRS2) and Glycogen Synthase Kinase-3 (GSK-3) may play important roles in the insulin signaling in the brain and AD processes (reviewed by Danièlle et al., 2018). We will also explore the relationship between molecular signals and glucoCEST signals in further studies.

A decreased concentration trend of the metabolites (Gln, mI, Cr + PCr, and Glu + Gln) from the bilateral hippocampus in AD rats was observed in this study. Although there were no statistical differences, we think the group differences may be observed with increased sample size. Decreased NAA/tCr and Glu/tCr ratios were also reported in previous animal studies with mice models of AD (von Kienlin et al., 2005). However, those changes were age-dependent index observed in 24-month-old animals instead of 12-month-old animals. Interestingly, an increased mI/tCr ratio was also reported in 20-month-old APP-PS1 mice (Marjanska et al., 2005) and amyloid-positive healthy elderly (Voevodskaya et al., 2016) in the cortex, though this is still a matter of controversy. We found a decreased mI in the bilateral hippocampus of an AD rat at 28 days after modeling, and it was related to GCE in this study. As a second messenger glucose isomer, mI is involved in glucose and insulin metabolism and promotes muscle glucose uptake (Chukwuma et al., 2016). We thus believe that decreased mI may mainly contribute to elevated MTRasym (0.9 ppm) signals and decreased GCE in AD rats. This is also abnormally increased in old age, and this may be as a compensation mechanism.

This study had some limitations: (1) the relatively small number of examined animals, which could impact the significance of our results; (2) the total scan time was too long, and in future studies, we need to use a more efficient method to increase brain glucose uptake; and (3) we used the Aβ25–35-induced AD rat instead of a double transgenic AD mouse model. Therefore, we will improve our experimental condition and enquire with a mouse head coil with a high channel in future studies.

## Conclusion

In this study, we found a reduced GCE signal of glucose in the whole brain and parietal cortex of AD rats *in vivo*, and reduced GCE in the total brain is associated with decreased concentrations of mI in the bilateral hippocampus of AD rats and control rats. Therefore, CEST-based MRI GCE can be a potentially valuable tool to explorer the early pathogenesis and pathological mechanisms of many diseases, such as diabetes-related AD and other kinds of dementia as well as neurodegenerative diseases.

## Data Availability Statement

The original contributions presented in the study are included in the article/supplementary material, further inquiries can be directed to the corresponding author/s.

## Ethics Statement

The animal study was reviewed and approved by the Ethics Committee of Animal Care and Welfare, Shantou University Medical College.

## Author Contributions

All authors listed have made a substantial, direct and intellectual contribution to the work, and approved it for publication.

## Conflict of Interest

ZS was employed by company Philips Healthcare. The remaining authors declare that the research was conducted in the absence of any commercial or financial relationships that could be construed as a potential conflict of interest.

## Abbreviations

CEST: Chemical Exchange Saturation Transfer
glucoCEST: glucose CEST
GCE: glucoCEST enhancement
MWM: Morris water maze
MTR: magnetization transfer ratio
DTI: Diffusion Tensor Imaging
MRS: magnetic resonance spectra
NAA: N-acetyl aspartate
Glu: glutamate
Cr: creatine
Cho: choline
mI: myo-Inosito.
